# Hybrid super electron donors – preparation and reactivity

**DOI:** 10.3762/bjoc.8.112

**Published:** 2012-07-03

**Authors:** Jean Garnier, Douglas W Thomson, Shengze Zhou, Phillip I Jolly, Leonard E A Berlouis, John A Murphy

**Affiliations:** 1 WestCHEM, Department of Pure and Applied Chemistry, University of Strathclyde, 295 Cathedral Street, Glasgow G1 1XL, United Kingdom

**Keywords:** aryl iodide, electron transfer, hybrid donors, reduction

## Abstract

Neutral organic electron donors, featuring pyridinylidene–imidazolylidene, pyridinylidene–benzimidazolylidene and imidazolylidene–benzimidazolylidene linkages are reported. The pyridinylidene–benzimidazolylidene and imidazolylidene–benzimidazolylidene hybrid systems were designed to be the first super electron donors to convert iodoarenes to aryl radicals at room temperature, and indeed both show evidence for significant aryl radical formation at room temperature. The stronger pyridinylidene–imidazolylidene donor converts iodoarenes to aryl anions efficiently under appropriate conditions (3 equiv of donor). The presence of excess sodium hydride base has a very important and selective effect on some of these electron-transfer reactions, and a rationale for this is proposed.

## Introduction

Alkenes that are substituted by four heteroatoms are notable for their ease of oxidation. Whereas tetrathiafulvalenes and analogues [1–5] have principally found widespread applications in materials science, tetraazaalkenes and related compounds are much more reactive and are of potential or actual interest as reagents in synthesis [6–33]. Among the tetraazaalkenes, those that are converted to aromatic molecules upon oxidation, e.g., tetraazafulvalenes **1** and **2**, are extremely reactive, and their electrochemical properties have been studied in some depth [13–16].

**Figure 1:**
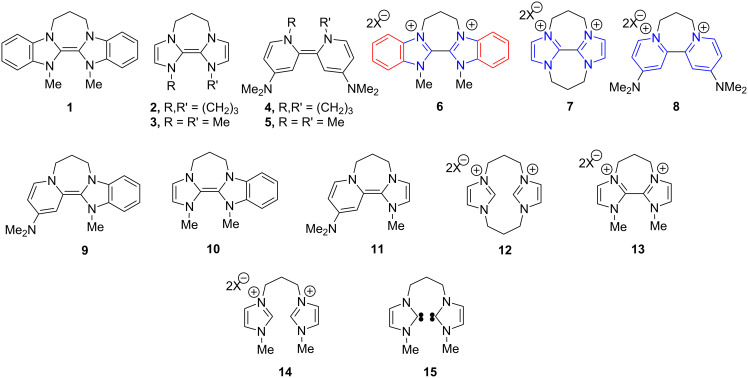
Super-electron-donors and related compounds.

Neutral organic donors that can reduce aryl halides have been termed “super-electron-donors”. Our recent research has examined the remarkable chemical reactivity of such donors **1** and **2** as well as the related electron-donors **4** and **5** (Figure 1), with organic substrates [17–27]. Benzimidazole-derived donor **1** converted aryl iodides to aryl radicals by transfer of a single electron at 110 °C [17], and was the first neutral organic ground-state molecule to achieve this. Later, the more powerful reagents **2** [18], **4** [20] and **5** [21] and related compounds [25] afforded aryl anions from the same substrates by transfer of two electrons at room temperature, and also cleaved selected sulfonamides [19], bis-sulfones [19], Weinreb amides [22], acyloin derivatives [24], triflate esters and triflamides [26]. Most recently, we announced the synthesis of the unstable compound **3** [16,27].

**Figure 2:**
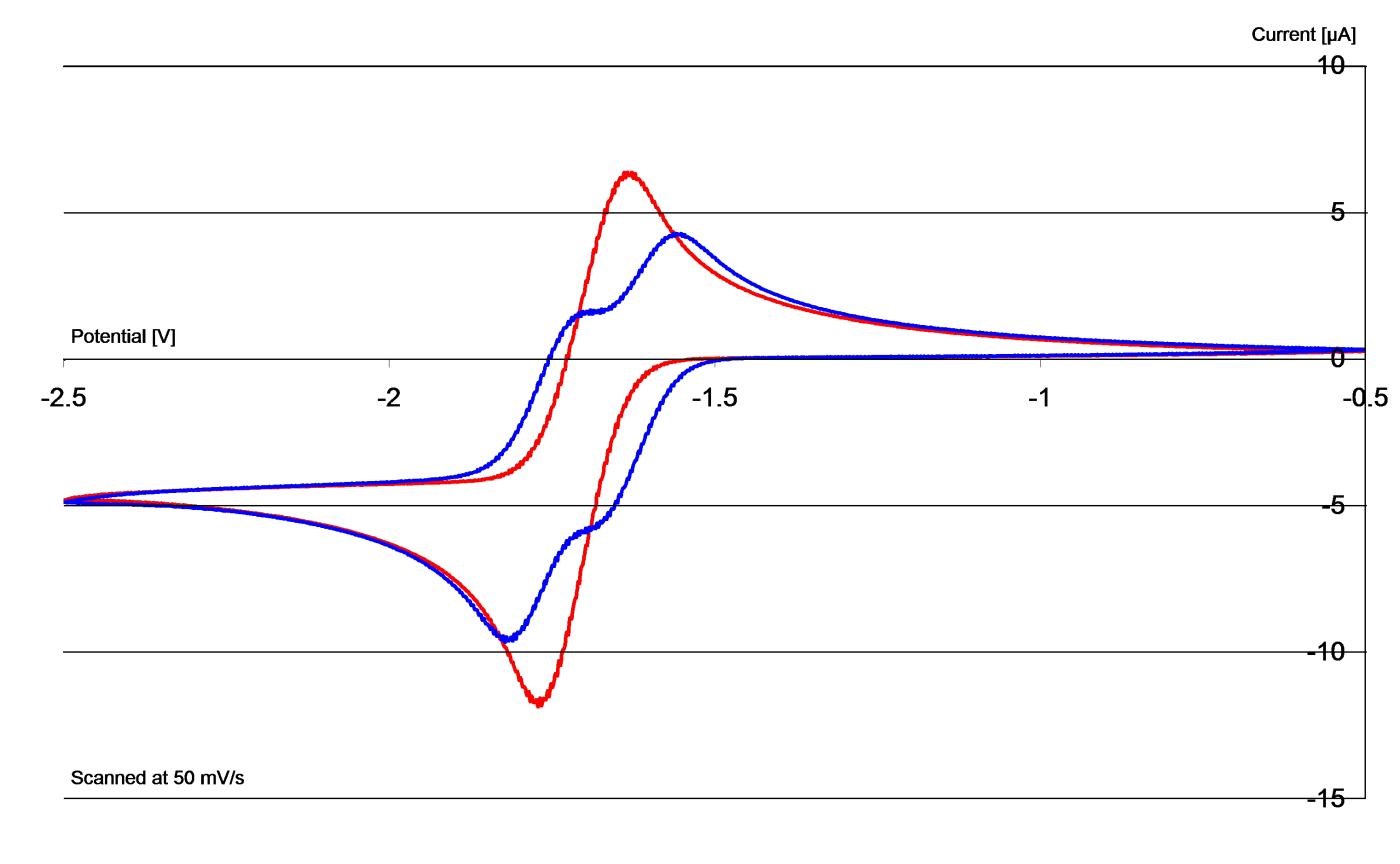
Cyclic voltammograms in DMF of **8**/**4** (red) and **21**/**11** (blue). Current plotted vs V (relative to Fc/Fc^+^ as standard).

Cyclic voltammetry (CV) studies showed that the more powerful donors, e.g., **2** [15–16,18] and **4** [19], lose their second electrons at almost the same potential as their first electron (Figure 2 includes the CV of **4**, showing a single two-electron redox wave, while Figure 4a includes that of **2**). The differential strengths of the donors (**2**, **4** versus **1**) correlated with the expected relative driving force resulting from aromatisation following the loss of electrons. In the case of the respective oxidised forms, i.e., the dications **6**–**8**, the newly aromatic rings are represented in blue in Figure 1, while the pre-existing aromatic rings in **6** are represented in red. The driving force for oxidation arising through aromatisation is greater for the imidazole- and pyridine-derived motifs **7** and **8**, which are associated with the strongest donors, than it is for the benzimidazole-derived motif, **6**, which marks a weaker donor.

To extend the capabilities of such reagents, we now set out to design the first neutral, organic ground-state donor that could, at room temperature, reduce aryl iodides to aryl radicals (thereby acting as a single-electron donor) as opposed to aryl anions. Hybrid organic electron donors incorporating a "stronger" donor component and a "weaker" component, e.g., **9** or **10** would be prime candidates, as the driving force for the loss of their first electron should exceed that for the loss of their second. The electrochemical properties of some hybrid imidazolium–benzimidazolium-derived donors have been reported [15]. For comparison, donor **11** is also of interest, although it features two "stronger" donor components. As indicated below, our work has found remarkable effects of excess base in reactions of some hybrid donors.

## Results and Discussion

Compounds **9**–**11** were adopted as targets for synthesis. Of these, **10** and **11** are imidazolylidenes derived from an imidazolium salt. Donors derived from imdazolium salts are highly reactive and unstable; CV studies in MeCN have shown [15] that two-electron reduction of **13**, bearing a single trimethylene bridge, which was intended to afford **3**, does not lead to a stable product; moreover, Taton and Chen [16] did not observe formation of **3** (by deprotonation of **14** in DMSO or by reduction of **13**), reporting instead the formation of bis-carbene **15**. In the recent synthesis of **3** [27], its decomposition was noted over a period of hours in ultradry conditions under argon. Within the series of imidazole-derived tetraazafulvalenes, Taton and Chen established [16] that the only member that remained stable on storage under inert conditions was the bis-trimethylene bridged donor **2**, and its greater stability was attributed to the two trimethylene tethers. As compounds **10** and **11** are derived from imidazolium precursors, we were keen to explore their reactivity.

**Scheme 1:**
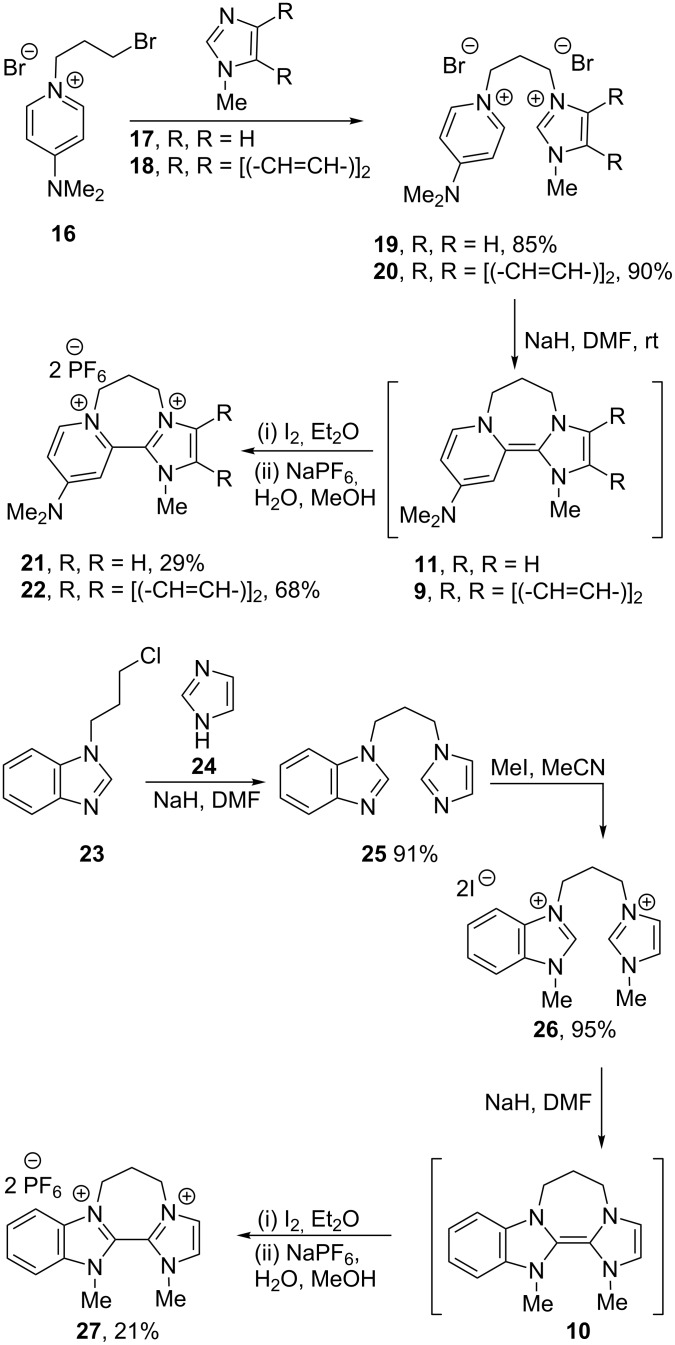
Preparation of the oxidised disalts.

The redox properties of the donors were first measured by cyclic voltammetry. Either the electron donors or their oxidized salts could, in principle, be used as a starting point for the CV studies; however, the oxidised disalts were routinely chosen as they can be conveniently weighed out under air, while the donors are extremely air-sensitive. The oxidized salts, derived from the donors, were prepared as shown in Scheme 1. Reaction of 1-(3-bromopropyl)-4-dimethylaminopyridinium bromide (**16**) [34] with *N*-methylimidazole (**17**) afforded disalt **19**. Deprotonation with NaH (15 equiv) in DMF then afforded the electron donor **11** in situ; this was reacted with iodine to afford the oxidised diiodide salt, and this was subjected to anion exchange to afford the bis(hexafluorophosphate) salt **21** for analysis. (Anion exchange to bis(hexafluorophosphate) salts was required since the iodide anions within diiodide salts would be electrochemically active in CV studies). To verify the intermediacy of **11**, its formation from **19** was repeated in DMF-*d*_7_, and the ^1^H and ^13^C NMR spectra of **11** were determined. The ^1^H NMR spectrum showed the characteristic upfield shift of proton signals for nonaromatic electron-rich donors.

Figure 2 shows the cyclic voltammogram of **21** (blue trace) and a comparison with **8** (X = PF_6_) (red trace). As seen, **21** undergoes reversible redox chemistry [*E*^1^_½_ (DMF) = −1.75 V, *E*^2^_½_ (DMF) = −1.63 V versus Fc/Fc^+^; this corresponds to *E*^1^_½_ (DMF) = −1.30 V, *E*^2^_½_ (DMF) = −1.18 V versus SCE]. The cyclic voltammogram, together with the NMR determination above, shows that **11** is a stable imidazole-derived donor (i.e., it does not decompose under the conditions used for its formation) [15], and so its capability as an electron donor was tested.

**Scheme 2:**
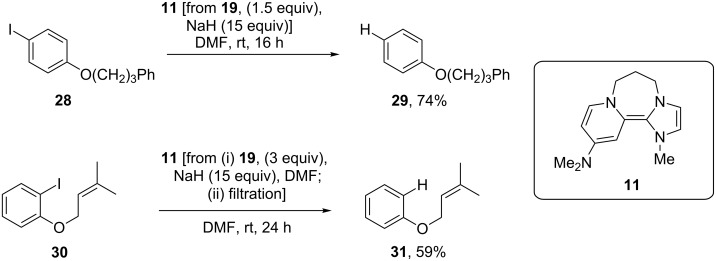
Reductive reactions of donor **11** [17–18].

To test reactivity, donor **11** was prepared in situ and treated with the substrates **28** and **30** at room temperature (Scheme 2). Simple substrate **28** [35] was added to **11**, prepared by adding disalt **19** (1.5 equiv) to excess sodium hydride (15 equiv). As expected, it behaved as a strong donor, affording **29** [20] in 74% yield. (A blank experiment, in which substrate **28** was treated at room temperature for 16 h with NaH in DMF, led to quantitative recovery of **28**). Substrate **30** [35] was designed to test whether a single electron or two electrons are transferred to an iodoarene; single electron transfer would afford an aryl radical that would undergo cyclisation efficiently [17], while two-electron transfer to afford an aryl anion would afford an aryl anion that would not cyclise in DMF as solvent [18]. The reaction with **30** was conducted under slightly different conditions than with **28**. Donor **11** was prepared by using disalt **19** (3 equiv) added to the excess sodium hydride (15 equiv), and the resulting donor solution was filtered to remove excess NaH before substrate **30** was added. (Previous experience had raised suspicion that the excess NaH could deprotonate the aliphatic side-chain of allyloxy substrates). The sole isolated product, i.e., the de-iodinated but uncyclised compound **31** (59%) [36], is consistent with **11** donating two electrons.

**Figure 3:**
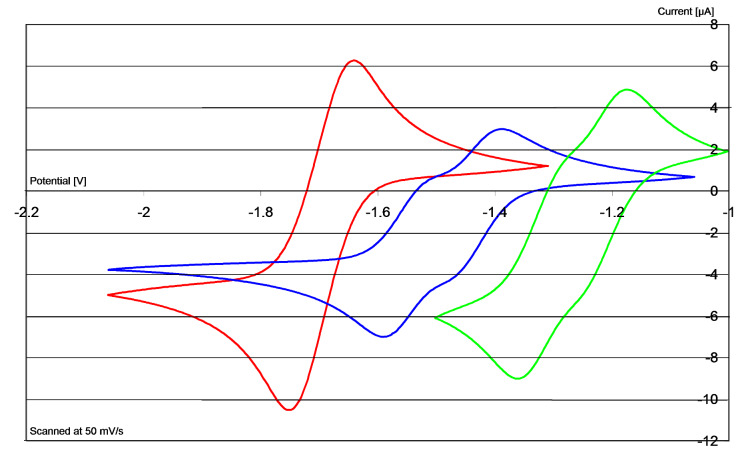
Cyclic voltammograms in DMF of **8/4** (red), **6/1** (green) and **22/9** (blue). Current plotted vs V relative to Fc/Fc^+^.

Donor **9** was prepared by a route analogous to that used for **11**, and was then oxidised and converted to the bis(hexafluorophosphate) salt **22**. Cyclic voltammetry, starting with its oxidised disalt **22** (Figure 3, blue trace) shows that its redox activity occurs as two separate steps at potentials intermediate between those for compounds **6** and **8**. [*E*^1^_½_ (DMF) = −1.54 V, *E*^2^_½_ (DMF) = −1.42 V versus Fc/Fc^+^; this corresponds to *E*^1^_½_ (DMF) = −1.09 V, *E*^2^_½_ (DMF) = −0.97 V versus SCE]. The cyclic voltammetry studies on oxidised disalt **22** show two reversible one-electron transitions on its reduction to donor **9**. The redox potential in the oxidation trace for the removal of the first electron from **9** shows that the molecule is not as strong a donor as **4**, while the transfer of the second electron occurs at a more negative potential than for the first electron from **1**.

**Scheme 3:**
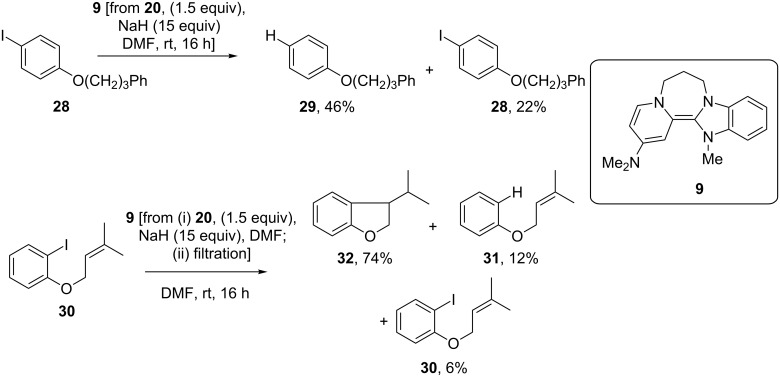
Reductive reactions of donor **9**.

In situ generation of **9** from **20** (1.5 equiv, Scheme 3) and reaction with iodoarenes **28** and **30** was again carried out at room temperature. As for the reactions with donor **11**, the excess NaH was filtered prior to the addition of substrate **30**. Reaction of iodoarene **28** led to an inseparable mixture of **29** and **28** in a 2:1 ratio; based on the mass recovered, this corresponded to **29** (46%) and **28** (22%). By comparison, reaction with aryl iodide **30**, again at room temperature, afforded a mixture of **32** [35], the product of aryl radical cyclization, (74%), together with recovered **30** (6%) and deiodinated but uncyclised product **31** (12%). This is the first observation of efficient aryl radical generation at room temperature from a super-electron-donor. For comparison, less than 1% yield of **32** was observed when repeating the reaction with donor **1**, also generated in situ. Hence **9** reacts at room temperature with iodoarenes and functions as the strongest known neutral organic ground-state one-electron donor to iodoarenes.

**Figure 4:**
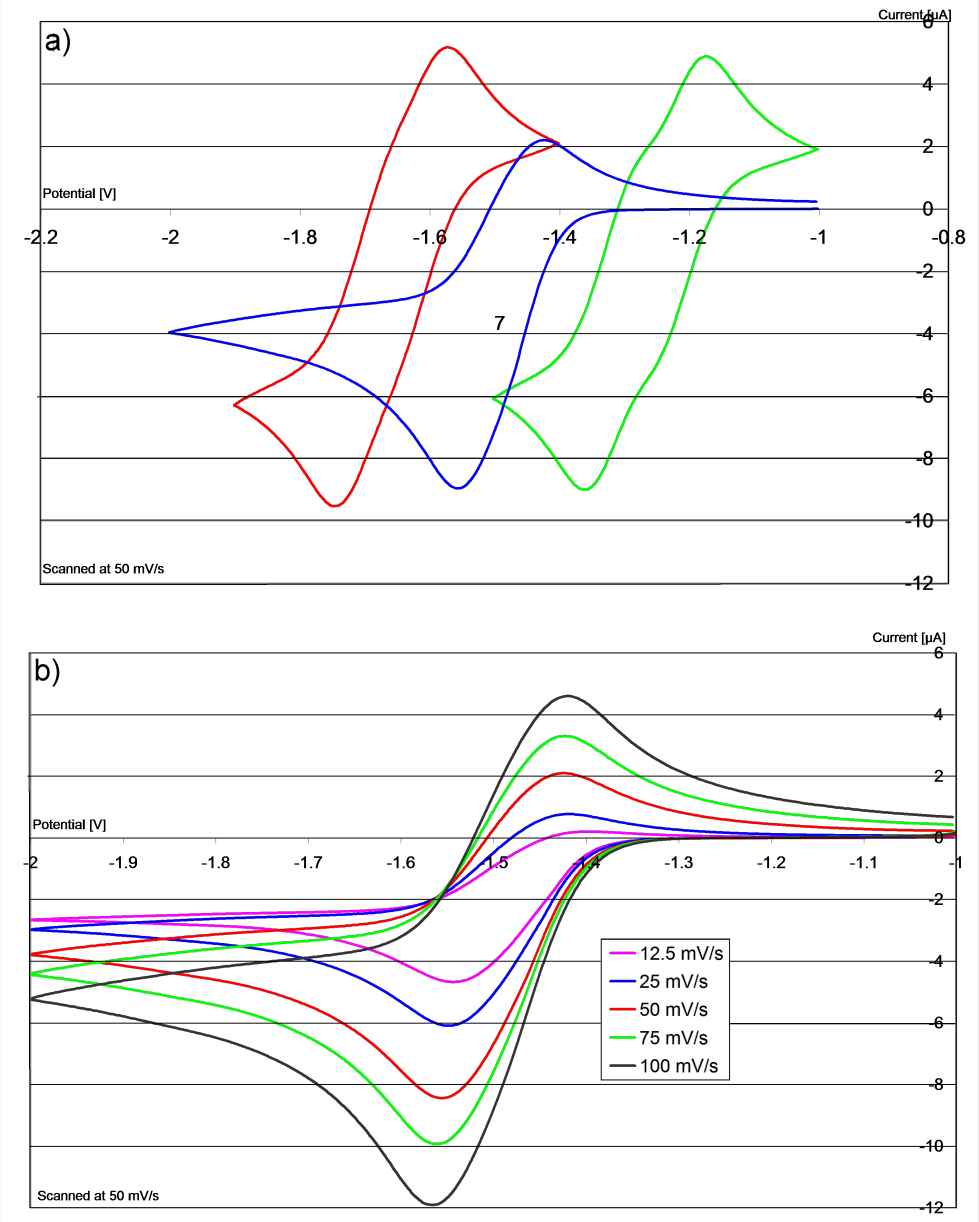
(a) c.v. in DMF of **7/2** (red), **6/1** (green) and **27/10** (blue); (b) c.v. in DMF of **27/10** at different scan rates. Current plotted vs V relative to Fc/Fc^+^.

Hybrid donor **10** was next prepared from the known chloropropylbenzimidazole **23** [37] (Scheme 1), then oxidised and converted to its bis(hexafluorophosphate) salt, **27**, for analysis. Cyclic voltammetry on **27** is shown in Figure 4a (blue trace), in comparison with salts **7** and **6**. Looking at the blue trace in Figure 4a, it is immediately clear that the oxidative sweep provides a very low current relative to the initial reductive sweep, suggesting decomposition of the reduced species on the timeframe of the CV studies. Repeating the experiment at different scan rates (Figure 4b) shows that at low scan speeds the effect is even more pronounced. Note that the CV traces reproduced by Ames et al. [15] demonstrate the same effect.

**Scheme 4:**
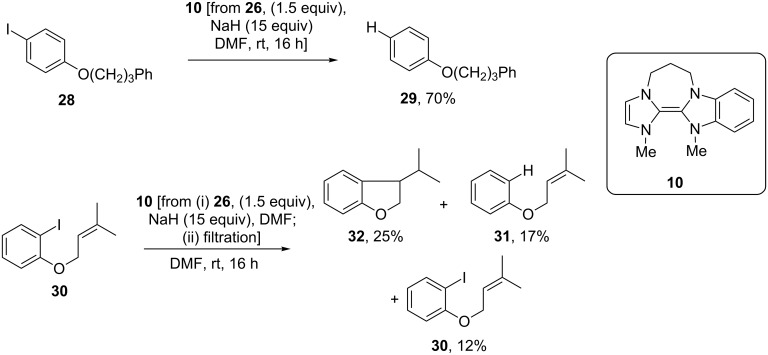
Use of hybrid donor **10** in reduction of iodoarenes.

This instability suggested that it should be difficult to obtain complete reaction when using **26** as a precursor of **10** in the preparative-scale reduction of aryl iodides. The standard two iodides **28** and **30** were tested under slightly different conditions, as mentioned above for donors **9** and **11**. Here a surprising outcome was seen. Complete reduction was observed for iodide **28**, affording a good isolated yield of **29** (70%) (Scheme 4). However, iodide **30** was reduced by **10** to give the products **32/31**/**/30** in a 2:1.4:1 ratio (^1^H NMR) with a poor overall recovery of 54%.

In both cases, the donor had been prepared in situ by reacting the precursor salt **26** (1.5 equiv) with excess sodium hydride (15 equiv). However, whereas **28** was simply added to the resulting mixture, which included residual excess base, the excess base had been removed by filtration prior to addition of **30**. This led us to question whether excess base could be helpful in such reactions and, if so, could the reported instability of other imidazole-based electron donors [15] also be addressed in the presence of base?

**Scheme 5:**
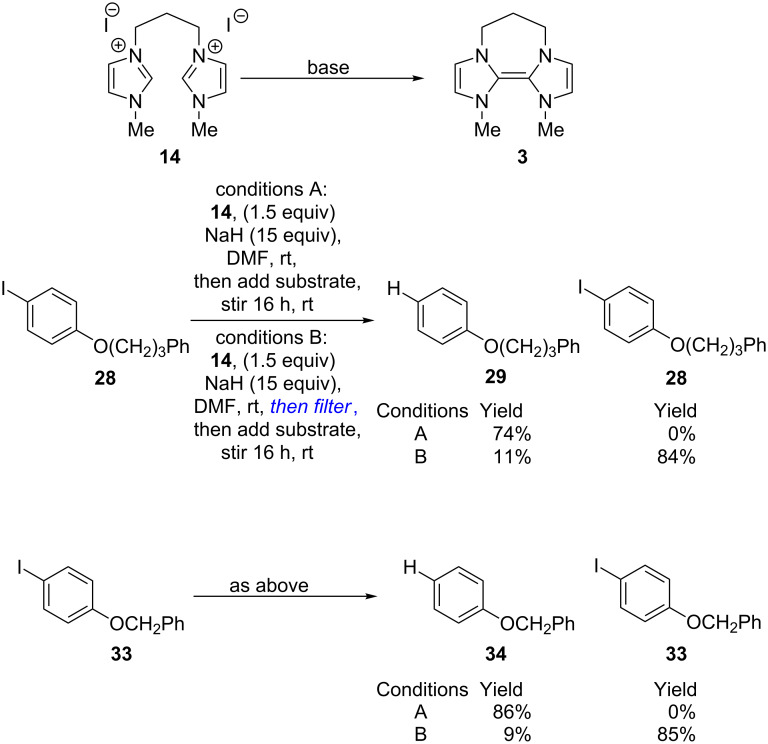
Reductive chemistry from disalt **15**.

To test this, mono-trimethylene precursor **14** [38], was prepared. This compound is the precursor of donor **3**. However, earlier CV studies to prepare **3** by reduction of **13** showed that **3** was not a stable compound, as discussed above [16,27]. Treating **14** with excess NaH, and then adding **28** to this reaction mixture pleasingly provided **29** (74%) exclusively (Scheme 5). However, repeating the same reaction, but filtering the excess NaH prior to addition of substrate **28** gave only 11% of reduced product **29**, together with starting substrate **28** (84%). The same outcome was seen with a second substrate, **33** [39]. In the presence of excess NaH, reduced product **34** was isolated in 86% yield, whereas when the substrate was added after removal of excess NaH, a lower yield of **34** (9%) was isolated, together with starting substrate **33** (85%).

**Scheme 6:**
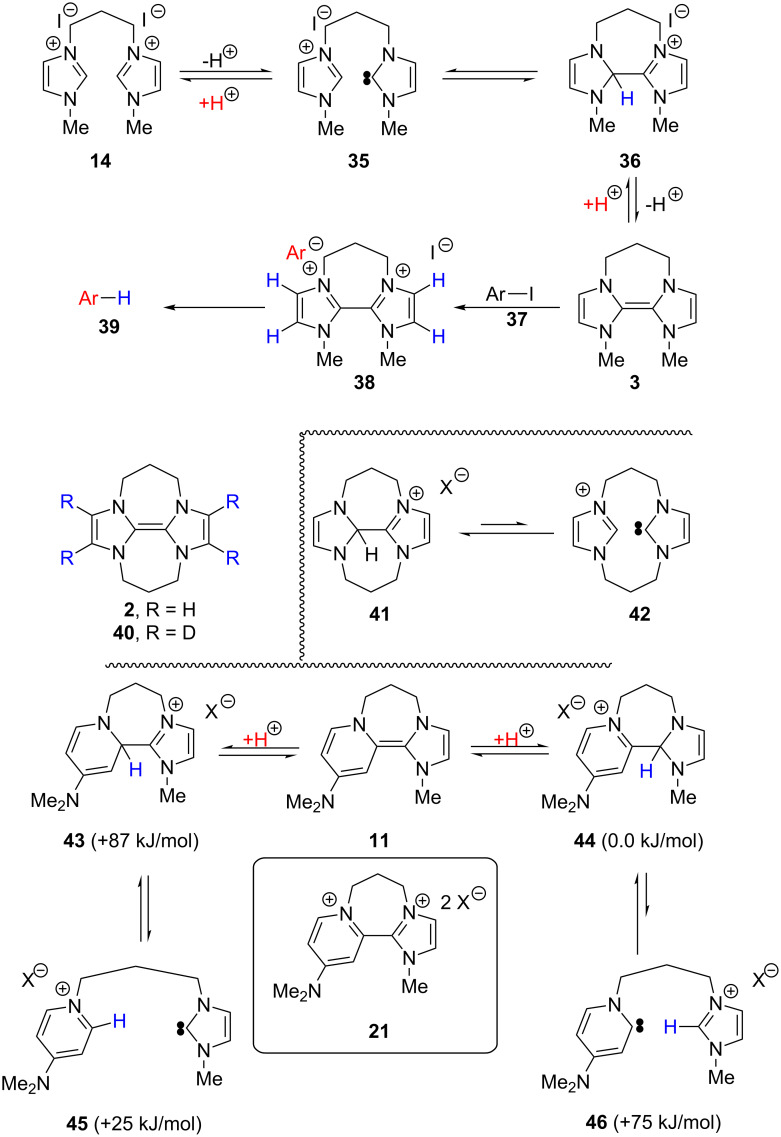
Rationalisation of effect of excess NaH base.

How can the base be assisting these reactions? Scheme 6 takes disalt **14** as an example.

Treatment of **14** with two equivalents of NaH would afford donor **3** as shown. This should then react with an iodoarene **37** to afford the dication salt **38** featuring an aryl anion and an iodide as counterions. In these circumstances, we suggest that the aryl anion can abstract a proton rapidly from the periphery of **38** to form reduced arene **39**, consistent with our previous studies on deprotonation of pyridinium salts [21]. However, **38** is a dication, and, to attain neutrality, could lose two protons. Compound **3** could be a strong base (in support of this, we have witnessed complete conversion of the analogous donor **2** to form **40** by rapid exchange in CD_3_CN as solvent; see Supporting Information File 1). We also note that in the previously reported electrochemical studies, irreversible behaviour was always observed in acetonitrile, consistent with a role of this solvent as a proton donor in the decomposition, whereas it was much more rarely reported in the much less acidic solvent DMF [14–15], and if the experiment were conducted with no excess of NaH base, **3** could itself act as a base. Protonation of **3** would afford **36**, capable of undergoing spontaneous fragmentation to **35** [40–43] thereby lowering the concentration of donor. However, excess sodium hydride can inhibit the protonation of **3** by competing for protons. (Notably, in earlier studies on the reversibility of formation of imidazoline-based donors, Liu and Lemal inhibited dissociation by adding KH as base [43]).

In the cyclic voltammetry case, **3** would be generated from disalt **38**. As **3** starts to be generated, it can deprotonate **38**, lowering the concentrations of **3** and therefore lowering the cathodic current in the CV, as observed for couple **27**/**10** in Figure 4.

It would then remain to explain why some imidazole-derived donors, e.g., **2** and **11**, apparently are not affected, or are much less affected by this problem. Protonation of **2** leads to **41**, and it is likely that the equilibrium fragmentation of this compound to **42** is less favourable than the fragmentation of **36** to **35** because of the restriction imposed by the second trimethylene bridge [16]. (Compound **41** has not previously been reported, but its existence is clear from its preparation here by deprotonation of **12** with one equivalent of NaH (see Supporting Information File 1). For protonated forms of other tetraaza donors, see [44–45]).

Compound **11** is likely to deprotonate dication **21** analogously to the previous examples. If **43** results from this protonation, then it should undergo easy fragmentation to **45**, featuring a pyridinium salt and an imidazolylidene, and in these circumstances, it would be difficult to understand why this electron-donor system works well. However, if isomeric compound **44** is the product of protonation, then its fragmentation to **46**, featuring an imidazolium salt and a pyridinylidene may well be relatively disfavoured. The pyridinylidene carbene in **46** should be less stabilised than the imidazolylidene carbene in **45**, since in the former case, the carbene is stabilised by only one neighbouring N atom. Keeping the inter-ring C–C bond in **44** could make reversion to donor **11** much more straightforward (than for **45**/**43**). This would then fit with our observations. Computational studies show indeed that **44** lies 87 kJ/mol below **43**, and so the preferred protonated form is **44**. Furthermore, fragmentation of **44** to **46** is indeed difficult, being uphill by 75 kJ/mol. This may explain why donor **11** is not significantly affected when excess base is absent.

## Conclusion

Hybrid organic super-electron-donors have been prepared, and their reactivity with aryl iodides tested. The donors show evidence for transfer of one electron or two electrons, dependent on their structure. Excess sodium hydride has a very beneficial effect on yields of products in certain cases, and a rationale for this has been proposed.

## Supporting Information

File 1 Experimental and computational details.
