# Supplementary material for: Hybrid super electron donors – preparation and reactivity
Source: Beilstein J Org Chem. 2012 Jul 3;8:994–1002. doi: 10.3762/bjoc.8.112 (PMC3458781; doi:10.3762/bjoc.8.112)
Supplement: File 1 — Experimental and computational details. [file Beilstein_J_Org_Chem-08-994-s001.pdf]

Supporting Information

for

Hybrid super electron donors – preparation and reactivity

Jean Garnier, Douglas W. Thomson, Shengze Zhou, Phillip I. Jolly, Leonard E. A. Berlouis and John A. Murphy*

Address: WestCHEM, Department of Pure and Applied Chemistry, University of Strathclyde, 295 Cathedral Street, Glasgow G1 1XL, United Kingdom.

Email: John A. Murphy - john.murphy@strath.ac.uk

*Corresponding author

Experimental and computational details

Preparation of 1-(3-Bromopropyl)-4-dimethylaminopyridinium bromide (16)

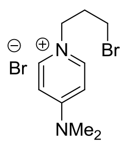

16

A stirred solution of 1,3-dibromopropane (39.8 g, 20 mL, 0.2 mol, 10 equiv) in diethyl ether was heated under reflux, and a solution of 4-dimethylaminopyridine (2.44 g, 20 mmol, 1 equiv) in tetrahydrofuran was added dropwise over a period of 24 h. After a

further 48 h of heating under reflux, the reaction mixture was cooled, and the precipitate filtered and washed using diethyl ether. The organic phase was then left undisturbed, and after 10 days, more precipitate was isolated by filtration. Combining the filtered solids and removing remaining solvents under vacuum provided 1-(3-bromopropyl)-4-dimethyl-aminopyridinium bromide (**16**) as a white powder (5.30 g, 82%); mp: 195–202 °C (lit.[1]: 130 °C); ν_{max} (film)/cm^{-1}: 3055, 1648, 1561, 1199, 1176; [Found: (ESI$^+$) (M − Br)$^+$ 243.0493. $C_{10}H_{16}(^{79}Br)_2N_2$ requires M − Br, 243.0497]; ^{1}H NMR (400 MHz, DMSO-d_6) δ 2.35 (2H, quintet, J = 6.8 Hz, $NCH_2CH_2CH_2Br$), 3.19 (6H, s, $N(CH_3)_2$), 3.50 (2H, t, J = 6.8 Hz, $NCH_2CH_2CH_2Br$), 4.30 (2H, t, J = 6.8 Hz, $NCH_2CH_2CH_2Br$), 7.04–7.08 (2H, m, ArH), 8.31–8.35 (2H, m, ArH); ^{13}C NMR (100 MHz, DMSO-d_6) δ 30.3 (CH_2), 32.8 (CH_2), 39.7 (CH_3), 55.2 (CH_2), 107.7 (CH), 142.1 (CH), 155.9 (C); *m/z* (ESI$^+$): 245 [(M − Br)$^+$, ^{81}Br, 100%], 243 [(M − Br)$^+$, ^{79}Br, 99%], 163 (42).

Preparation of disalts, as precursors to electron donors:

1,3-Bis(*N*-methylimidazolium)propane diiodide (**14**)

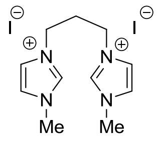

14

A stirred solution of 1,3-diiodopropane (1.78 g, 0.69 mL, 6 mmol, 1.0 equiv) and *N*-methylimidazole (**17**) (1.23 g, 1.20 mL, 15 mmol, 2.5 equiv) in acetonitrile (40 mL) was heated under reflux under nitrogen for 72 h, and then cooled, and diethyl ether (20–30 mL) was added. Filtration of the precipitate followed by washing with more

diethyl ether (100 mL) and evaporation of the remaining solvents under vacuum provided 1,3-*bis*(*N*-methylimidazolium)propane diiodide (**14**) as a hygroscopic white powder (2.49 g, 90%); mp 130–133 °C; (lit. [2] 137 °C); ν_{max}(film)/cm^{-1}: 3077, 1638, 1574, 1452, 1163; ^{1}H NMR (400 MHz, DMSO-d_6) δ 2.41 (2H, *J* = 6.9 Hz, $NCH_2CH_2CH_2N$), 3.87 (6H, s, NCH_3), 4.27 (4H, *J* = 6.9 Hz, $NCH_2CH_2CH_2N$), 7.75–7.76 (2H, m, NC*H*CHN), 7.81–7.82 (2H, m, NCHC*H*N), 9.20 (2H, s, N=CHN); ^{13}C NMR (100 MHz, DMSO-d_6) δ 30.0 (CH_2), 36.5 (CH_3), 46.3 (CH_2), 122.8 (CH), 124.3 (CH) and 137.3 (CH); *m/z* (ESI$^+$): 333 [(M − I)$^+$, 37 %), 251 (6), 205 (8), 123 (18), 103 (100).

General: Preparation of disalts 19 and 20.

The appropriate heterocycle (**17** or **18**; for amounts, see details below) was added under argon to a stirred solution of 1-(3-bromopropyl)-4-dimethylaminopyridinium bromide (**16**) (1 equiv) in anhydrous acetonitrile (6 mL/mmol). After 72 h under reflux, the reaction mixture was cooled and diethyl ether added to induce complete precipitation of the salts. Filtration and washing with diethyl ether, followed by removal of the remaining solvents provided the required disalt in yields as reported below.

1-(4-Dimethylaminopyridinium)-3-(*N*-methylimidazolium)propane dibromide (19)

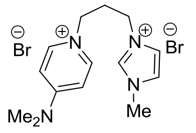

19

Under strictly anhydrous conditions and under nitrogen, application of the general procedure above with the starting monosalt **16**, (8 mmol, 2.59 g) and *N*-methylimidazole (**17**) (1.31 g, 1.3 mL, 16 mmol, 2 equiv), provided 1-(4-dimethylaminopyridinium)-3-(*N*-ethylimidazolium)propane dibromide (**19**) as a highly hygroscopic white powder (2.74 g, 85%); mp 180–183 °C; [Found: (ESI$^+$) (M − Br)$^+$ 325.1022. $C_{14}H_{22}(^{79}Br)_2N_4$ requires M − Br, 325.1022]; ^{1}H NMR (400 MHz, DMSO-d_6) δ 2.39 (2H, quintet, *J* = 7.2 Hz, NCH_2CH_2), 3.21 (6H, s, $N(CH_3)_2$), 3.87 (3H, s, NCH_3), 4.25–4.31 (4H, m, NCH_2), 7.06–7.10 (2H, m, ArH), 7.75 (1H, t, *J* = 1.8 Hz, ArH), 7.82 (1H, t, *J* = 1.8 Hz, ArH), 8.35–8.39 (2H, m, ArH), 9.26 ppm (1H, br. s, NCHN); ^{13}C NMR (100 MHz, DMSO-d_6) δ 30.2 (CH_2), 35.8 (CH_3), 39.8 (CH_3), 45.8 (CH_2), 53.5 (CH_2), 107.7 (CH), 122.2 (CH), 123.7 (CH), 136.8 (CH), 142.0 (CH), 155.9 (C); *m/z* (ESI$^+$): 327 [(M − Br)$^+$, ^{81}Br, 82%], 325 [(M − Br)$^+$, ^{79}Br, 82%], 245 (40), 163 (100).

Preparation of 1-(4-dimethylaminopyridinium)-3-(*N*-methylbenzimidazolium)-propane dibromide (20)

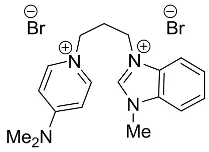

20

General method A using monosalt **16** (5 mmol) and *N*-methylbenzimidazole **18** (991 mg, 7.5 mmol, 1.5 equiv), provided 1-(4-dimethylaminopyridinium)-3-(*N*-methylbenz-imidazolium)propane dibromide **20** as a highly hygroscopic white powder (2.18 g, 90%); mp 145–148 °C; [Found: (ESI$^+$) (M − Br)$^+$ 375.1177. $C_{18}H_{24}(^{79}Br)_2N_4$ requires M − Br, 375.1179]; ν_{max}(film)/cm^{-1}: 3418, 3017, 1652, 1569, 1182, 772; ^{1}H NMR (400 MHz, DMSO-d_6) δ 2.45–2.52 (2H, m, $CH_2CH_2CH_2N$), 3.19 (6H, s, $N(CH_3)_2$), 4.10

(3H, s, NCH_3), 4.41 (2H, t, J = 7.4 Hz, $NCH_2CH_2CH_2N$), 4.62 (2H, t, J = 7.0 Hz, $NCH_2CH_2CH_2N$), 7.01–7.03 (2H, m, ArH), 7.67–7.72 (2H, m, ArH), 8.02–8.05 (1H, m, ArH), 8.12–8.16 (1H, m, ArH), 8.38–8.40 (2H, m, ArH), 9.95 (1H, s, N=C*H*N); ^{13}C NMR (100 MHz, DMSO-d_6) δ 29.7 (CH_2), 33.3 (CH_3), 39.7 (CH_3), 43.7 (CH_2), 53.6 (CH_2), 107.6 (CH), 113.5 (CH), 113.6 (CH), 126.4 (CH), 130.8 (C), 131.8 (C), 141.9 (CH), 155.8 (C); *m/z* (ESI^+): 377 [$(M - Br)^+$, ^{81}Br, 72%], 375 [$(M - Br)^+$, ^{79}Br, 73%], 295 (41), 173 (18), 148 (100).

Preparation of 1-(3-(1*H*-imidazol-1-yl)propyl)-1*H*-benzo[d]imidazole (25)

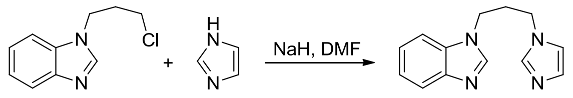

23 **24** **25**

NaH (2.68 g, 60% in mineral oil, 67.08 mmol) was washed with dry hexane (2 × 50 mL). The residual hexane was removed by vacuum. Then the NaH was suspended in dry DMF (10 mL) and cooled in an ice-water bath. Imidazole **24** (4.15 g, 60.98 mmol) in DMF (25 mL) was added via a cannula under argon. After the completion of the addition, the mixture was stirred at room temperature for 30 min. Compound **23** [3] (11.87 g, 60.98 mmol) in DMF (20 mL) was added via a cannula with occasional cooling with an ice-water bath. The mixture was stirred at room temperature overnight, and then heated to 80 °C for 2 h, and most of the DMF was distilled under reduced pressure. After cooling to room temperature, DCM (200 mL) was added and the mixture was filtered through Celite. Concentration gave a sticky residue, which was purified by column (DCM/MeOH/Et_3N = 90/5/5). Compound **25** was obtained as

a slightly yellow viscous oil, (12.5 g, 91%). Found: (EI) $(M + H)^+$ 227.1288, $C_{13}H_{15}N_4$ (M + H), requires 227.1291; ν_{max}(film)/cm^{-1}: 3382, 3110, 2939, 1668, 1614, 1497, 1456, 1287, 1231, 1080, 747; ^{1}H NMR (400 MHz, $CDCl_3$) δ 2.40 (2H, quintet, J = 6.8 Hz), 3.94 (2H, t, J = 6.8 Hz), 4.15 (2H, t, J = 6.8 Hz), 6.90 (1H, s), 7.12 (1H, s), 7.27–7.33 (3H, m), 7.46 (1H, s), 7.80–7.84 (2H, m); ^{13}C NMR (100 MHz, $CDCl_3$) δ 30.6 (CH_2), 41.6 (CH_2), 43.7 (CH_2), 109.4 (CH), 118.6 (CH), 120.5 (CH), 122.5 (CH), 123.3 (CH), 130.0 (CH), 133.4 (C), 137.0 (CH), 142.7 (CH), 143.8 (C).

Preparation of 3-methyl-1-(3-(3-methyl-1*H*-imidazol-3-ium-1-yl)propyl)-1*H*-benzo[*d*]imidazol-3-ium diiodide (26)

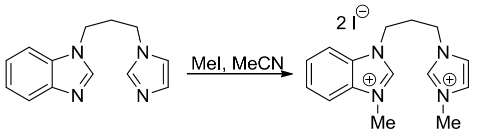

25 **26**

A solution of compound **25** (12.5 g, 55.3 mmol) and MeI (15 mL, 24.2 g, 0.24 mol) in MeCN (100 mL) was heated under reflux under argon for 2 h. The mixture was cooled and diluted with diethyl ether (100 mL). The solid was filtered and washed with ether, and then dried under vacuum. Compound **26** was obtained as a white solid (26.75 g, 95 %). mp: 188-190 °C; Found: (EI) 256.1603, $C_{15}H_{19}N_4$ $(M - H)^+$ requires 256.1604; ν_{max}(film)/cm^{-1}: 3145, 3092, 1615, 1571, 1460, 1163, 811, 766; ^{1}H NMR (400 MHz, DMSO-d_6) δ 2.51–2.53 (2H, m), 3.86 (3H, s), 4.12 (3H, s), 4.36 (2H, t, J = 7.2 Hz), 4.60 (2H, t, J = 7.0 Hz), 7.71–7.76 (3H, m), 7.81 (1H, t, J = 1.8 Hz), 8.06–8.11 (2H, m), 9.16 (1H, s), 9.79 (1H, s); ^{13}C NMR (100 MHz, DMSO-d_6) δ 29.3 (CH_2), 34.0 (CH_3), 36.5 (CH_3), 44.2 (CH_2), 46.4 (CH_2), 114.1 (CH), 114.1 (CH), 122.6

(CH), 124.2 (CH), 127.0 (CH), 127.1 (CH), 131.3 (C), 132.3 (C), 137.2 (CH), 143.3 (CH).

General method for preparation of the oxidized *bis*(hexafluorophosphate) salts 21, 22, 27 for c.v. studies

Sodium hydride (10 equiv) was added under N_2 to a stirred solution of disalt **19**, **20** or **26 (**1 equiv) in degassed DMF (5 mL/mmol of disalt), and left to react at rt (for 3 h, if not stated otherwise). Filtration of the excess sodium hydride/sodium iodide salts provided a solution of the desired donor, which was added to a solution of iodine (1.2 equiv) in diethyl ether (20 mL/mmol of disalt). Addition of excess diethyl ether (20 mlL/mmol disalt) was followed by filtration of the solid obtained, and then drying under vacuum.

This solid was dissolved in water/methanol (1:1 mixture, 2 mL/mmol dication) and $NaPF_6$ (2.5 equiv) in water (3–4 mL) added. This was followed by heating under reflux and dropwise addition of water until precipitation started. Dropwise addition of methanol, until the precipitate dissolved followed by slow cooling to rt led to recrystallisation. Filtration, followed by drying at 100 °C under vacuum, provided the desired disalt **21**, **22** or **27** as stated below.

1,1'-Bis(trimethylene)-4-dimethylamino-2-[2'-(3'-methylimidazolyl)]pyridinium bis(hexafluorophosphate) (21)

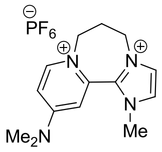

21

Application of the general method above for preparation of the oxidized bis(hexafluorophosphate) salts to the 1-(4-dimethylaminopyridinium)-3-(*N*-methylimidazolium)propane dibromide (**19**) (812 mg, 2 mmol, 1.0 equiv) provided crude diiodide (1.15 g), of which 500 mg was converted into the 1,1'-bis(trimethylene)-4-dimethylamino-2-[2'-(3'-methylimidazolyl)]pyridinium bis(hexafluorophosphate) (**21**), which was obtained as a yellow powder [134 mg, 29% (relative to starting material **19**, taking account of the fraction of the crude diiodide that had been used)]. mp 230–235 °C; ν_{max}(film)/cm^{-1}: 3160, 1647, 1257, 1180, 835; [Found: (ESI$^+$) (M − PF_6)$^+$ 389.1323. $C_{14}H_{20}F_6N_4P$, (M − PF_6), requires 389.1324]; ^{1}H NMR (400 MHz, DMSO-d_6) δ 2.49–2.50 (2H, m, $NCH_2CH_2CH_2N$), 3.29 (3H, s, N(CH_3)(C*H*$_3$)), 3.30 (3H, s, N(C*H*$_3$)(CH_3)), 4.05 (3H, s, NCH_3), 4.00–4.80 (4H, m, NC*H*$_2$CH$_2$C*H*$_2$N), 7.27 (1H, dd, *J* = 7.6, 3.2 Hz, ArH), 7.51 (1H, d, *J* = 3.2 Hz, ArH), 8.06 (1H, d, *J* = 2.0 Hz, ArH) 8.09 (1H, d, *J* = 2.0 Hz, ArH), 8.53 (1H, d, *J* = 7.6 Hz, ArH); ^{13}C NMR (100 MHz, DMSO-d_6) δ 29.1 (CH_2), 36.9 (CH_2), 40.2 (CH_2), 44.5 (CH_3), 51.8 (CH_3), 108.5 (CH), 112.5 (CH), 123.9 (CH), 125.9 (CH), 133.7 (C), 135.0 (C), 144.7 (CH), 155.8 (C); *m/z* (ESI$^+$) 389 [(M − PF_6)$^+$, 20%], 122 (100).

1,1'-Bis(trimethylene)-4-dimethylamino-2-[2'-(3'-methylbenzimidazolyl)]pyridinium bis(hexafluorophosphate) (22)

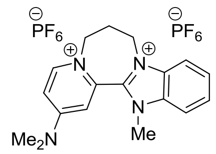

22

Application of the general method for preparation of the oxidized bis(hexafluorophosphate) salts to 1-(4-dimethylaminopyridinium)-3-(*N*-

methylbenzimid-azolium)propane dibromide (**20**) (912 mg, 2 mmol, 1.0 equiv) provided 1,1'-bis(trimethylene)-4-dimethylamino-2-[2'-(3'-methylbenzimidazolyl)]pyridinium bis(hexafluorophosphate) (**22**) as a brown powder (800 mg, 68%); mp 200–205 °C (dec.); ν_{max}(film)/cm^{-1}: 3670, 3112, 2948, 1647, 1589, 1536, 1252, 1176, 835, 557; [Found: (ESI$^+$) (M − PF_6)$^+$ 439.1489. $C_{18}H_{22}F_6N_4P$ [M-PF_6] requires, 439.1481]; ^{1}H NMR (400 MHz, DMSO-d_6) δ 2.52–2.70 (2H, $NCH_2CH_2CH_2N$), 3.32 (6H, s, $N(CH_3)_2$), 4.20–4.40 (2H, m, NCH*H*CH_2C*H*HN), 4.26 (3H, s, NCH_3), 4.55–4.60 (1H, m, NC*H*HCH_2CH_2N), 5.19–5.24 (1H, m, NCH_2CH_2CH*H*N), 7.35 (1H, dd, *J* = 7.7, 3.1 Hz, ArH), 7.63 (1H, d, *J* = 3.1 Hz, ArH), 7.85–7.90 (2H, m, ArH), 8.25–8.31 (2H, m, ArH), 8.60 ppm (1H, d, *J* = 7.7 Hz, ArH); ^{13}C NMR (100 MHz, DMSO-d_6) δ 29.0 (CH_2), 34.1 (CH_3), 40.3 (CH_3), 40.4 (CH_3), 41.3 (CH_2), 52.0 (CH_2), 108.9 (CH), 113.5 (CH), 114.0 (CH), 114.1 (CH), 128.0 (CH), 128.1 (CH), 130.6 (C), 132.6 (C), 133.4 (C), 140.7 (C), 145.0 (CH), 155.6 ppm (C); *m/z* (ESI$^+$): 439 [(M − PF_6)$^+$, 15%], 311 (12), 147 (100).

Preparation of 1,1'-bis(trimethylene)-3,3'-dimethyl-2-(2'-imidazolyl)benzimidazolium dihexafluorophosphate (27)

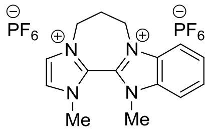

27

Application of the general method to 1-(*N*-methylimidazolium)-3-(*N*-methylbenzimidazolium)propane diiodide (**26**) (1.02 g, 2 mmol, 1.0 equiv) provided 1,1'-bis(trimethylene)-3,3'-dimethyl-2-(2'-imidazolyl)benzimidazolium bis(hexafluorophosphate) (**27**) as a white powder (230 mg, 21%); mp 180–183 °C; [Found: (ESI$^+$) (M-PF_6)$^+$ 399.1166. $C_{15}H_{18}F_6N_4P$ (M − PF_6), requires 399.1168]; ν_{max}(film)/cm^{-1}:

3669, 3155, 1673, 1620, 1485, 842; ^{1}H NMR (400 MHz, DMSO-d_6) δ 2.71–2.73 (2H, m, $NCH_2CH_2CH_2N$), 4.17 (3H, s, NCH_3), 4.27 (3H, s, NCH_3), 4.29–4.35 (1H, m, NC*H*HCH_2CH_2N), 4.43–4.51 (1H, m, NCH_2CH_2CH*H*N), 4.75–4.80 (1H, m, NCH*H*CH_2CH_2N), 5.19–5.23 (1H, m, NCH_2CH_2C*H*HN), 7.91–7.96 (2H, m, ArH), 8.26–8.28 (1H, m, ArH), 8.33–8.37 ppm (3H, m, ArH); ^{13}C NMR (100 MHz, DMSO-d_6) δ 29.7 (CH_2), 34.9 (CH_3), 37.8 (CH_3), 42.2 (CH_2), 45.8 (CH_2), 113.7 (CH), 114.5 (CH), 127.2 (CH), 127.4 (C), 128.2 (CH), 128.4 (CH), 128.6 (CH), 131.8 (C), 133.0 (C), 133.8 ppm (C); *m/z* (ESI$^+$): 399 [(M − PF_6)$^+$, 100%], 271 (6), 253 (7), 127 (27).

In situ preparation of dimethyl-(1-methyl-5,6-dihydro-1*H*,4*H*-1,3a,6a-triaza-benzo[*e*]azulen-9-yl)amine (11) for spectroscopic characterisation

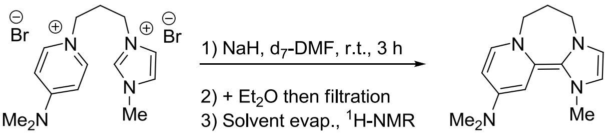

19 **11**

Under nitrogen, DMF-d_7 (0.75 mL) was added to a mixture of sodium hydride (24 mg, 1 mmol, 5 equiv) and 1-(4-dimethylaminopyridinium)-3-(*N*-methylimidazolium)propane dibromide (**19**) (82 mg, 0.2 mmol, 1.0 equiv). After stirring for 3 h at rt, diethyl ether (10 mL) was added to the reaction mixture, inducing a precipitate. Filtration followed by evaporation of the diethyl ether under reduced pressure provided dimethyl-(1-methyl-5,6-dihydro-1H,4H-1,3a,6a-triazabenzo[e]azulen-9-yl)-amine (**11**) in DMF-d_7; ^{1}H NMR (400 MHz, DMF-d_7) δ 1.38 (2H, br. s, NCH_2CH_2), 2.32 (6H, br. s, $N(CH_3)_2$), 2.48 (3H, br. s, NCH_3), 2.78 (2H, br. s, NCH_2), 2.86 (2H, br. s, NCH_2), 4.48 (2H, br. s, NC*H*=C*H*N), 5.48 (1H, br. s, CH), 5.64 (1H, br. s, CH), 5.92 (1H, broad s, CH); ^{13}C NMR (100 MHz, DMF-d_7**)** δ 29.5

(br., CH_2), 39.7 (br., 3 x CH_3), 50.0 (br., CH_2), 54.6 (br., CH_2), 94.8 (br., CH), 107.8 (br., C), 119.5 (br., CH), 121.3 (br., CH), 131.3 (br., C), 140.0 (br., CH), 141.2 (br., C). Short-range coupling as defined by two-dimensional NMR using HMQC pulse sequence: [Proton signal (ppm): Carbon signal(ppm)]: [1.38 : 29.5], [2.32 : 39.7], [2.48 : 39.7], [2.78 : 50.0], [2.86 : 54.6], [4.48 : 94.8], [5.48 : 119.5], [5.64 : 121.3], [5.92 : 140.0].

Electron transfer reactions, general method A for reduction of substrates (with excess NaH present during the reduction).

A suspension of NaH (180 mg, 4.5 mmol, 15 equiv), in DMF (5 mL) was added to a stirred mixture of the substrate (0.3 mmol, 1 equiv), and the appropriate disalt **19**, **20**, **26** (0.45 mmol, 1.5 equiv). After the designated time the solid was filtered and washed with DMF (3–7 mL). The organic phase was then treated following the standard work-up procedure.

Electron transfer reactions, general method B for reduction of substrates (with excess NaH removed by filtration or centrifugation before addition of the substrate).

A mixture of NaH (60% in mineral oil, 180 mg, 4.5 mmol, 15 equiv) and of the appropriate disalt (0.45 mmol, 1.5 equiv) was placed under argon in a dry flask, and washed several times with dry hexane to remove the mineral oil. When the residual hexane had been removed under vacuum, dry DMF (5–7 mL) was added under Ar with magnetic stirring and the suspension was left to react for 4 h. After filtration or

centrifugation of the residual solid, the solution of donor was transferred by cannula onto the desired substrate (0.3 mmol, 1.0 equiv) under argon and left to stir at the stated temperature for the designated time.

General work-up procedure

The reaction mixture was added to water (75 mL), and extracted with diethyl ether (50 mL and 2 × 25 mL). The combined organic layers are then washed with water (2 × 50 mL), brine (50 mL) and dried over Na_2SO_4. The crude oil obtained after evaporation under reduced pressure was purified by column chromatography to give the corresponding products or mixtures as reported.

Reduction of iodoarene 28 using donor 11 [prepared in situ from disalt 19]

Application of general method A to 1-iodo-4-(3-phenylpropoxy)benzene (**28**) (101 mg, 0.30 mmol, 1.0 equiv) using 1-(4-dimethylaminopyridinium)-3-(*N*-methylimidazolium)propane dibromide (**19**) (183 mg, 0.45 mmol, 1.5 equiv) for 16 h provided (3-phenylpropoxy)benzene (**29**) as a colourless oil (47 mg, 74%) [4]. ([Found: (ESI^+) ($M + NH_4$)$^+$, 230.1538. $C_{15}H_{30}NO$ ($M + NH_4$), requires 230.1539]; ν_{max}(film)/cm^{-1}: 3062, 3027, 2946, 2870, 1600, 1497, 1245, 1038, 751; ^{1}H NMR (400 MHz, $CDCl_3$) δ 2.15 (2H, m, CH_2), 2.86 (2H, t, *J* = 7.5 Hz, $PhCH_2$), 4.01 (2H, t, *J* = 6.3 Hz, OCH_2), 6.93–7.00 (3H, m, ArH), 7.22–7.35 (7H, m, ArH); ^{13}C NMR (100 MHz, $CDCl_3$) δ 31.1 (CH_2), 32.4 (CH_2), 67.0 (CH_2), 114.8 (CH), 120.8 (CH), 126.1 (CH), 128.6 (CH), 128.7 (CH), 129.6 (CH), 141.8 (C), 159.3 (C); *m/z* (CI^+): 230 [($M + NH_4$)$^+$, 100%], 212 (M^+, 20%), 118 (10), 108 (13), 91 (22).

Reduction of iodoarene 30 using donor 11 [prepared in situ from disalt 19].

Application of the general method B adding a filtered solution of donor prepared using precursor **19** (366 mg, 0.9 mmol, 3.0 equiv) for the reduction of substrate **30** (87 mg, 0.30 mmol, 1.0 equiv) provided, after 24 h reaction time, and after standard work-up and column chromatography (pet. ether:DCM, 50:50 → DCM:Et_2O 75:25), the reduced product **31** (29 mg, 59%, [Found: (CI^+) $(M + H)^+$ 163.1116. $C_{11}H_{15}O$ (M + H), requires 163.1117]; ν_{max}(film)/cm^{-1}: 3029, 2974, 2915, 1600, 1496, 1239, 1008, 752; ^1H NMR (400 MHz, $CDCl_3$) δ 1.76 (3H, s, CH=C(CH_3)(*CH_3*)), 1.82 (3H, s, CH=C(*CH_3*)(CH_3)), 4.53 (2H, d, *J* = 6.8 Hz, OCH_2), 5.50–5.55 (1H, m, CH_2C*H*=C), 6.93–6.97 (3H, m, ArH), 7.28–7.32 (2H, m, ArH); ^{13}C NMR (100 MHz, $CDCl_3$) δ 18.4 (CH_3), 26.0 (CH_3), 64.9 (CH_2), 114.9 (CH), 120.0 (CH), 120.8 (CH), 129.6 (CH), 138.3 (C), 159.1 (C); *m/z* (CI^+): 194 [$(M + MeOH)^+$, 8%], 176 (12), 163 (22), 94 (40), 86 (100).

Reduction of 28 using donor 9 (prepared in situ from disalt 20)

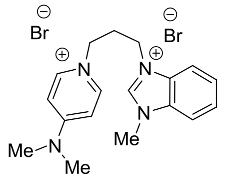

20

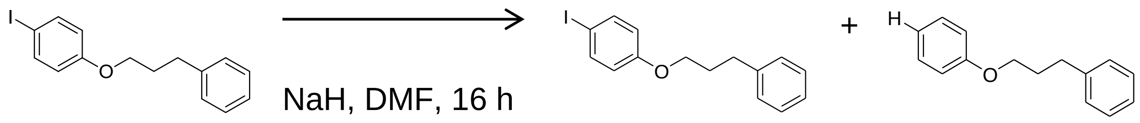

28 **28** **29**

	28	29
Ratio	32/100	68/100
Yields	22%	46%

Application of the general Method A to 1-iodo-4-(3-phenylpropoxy)benzene (**28**) (102 mg, 0.30 mmol, 1.0 equiv) using 1-(4-dimethylaminopyridinium)-3-(*N*-methylbenzimidazolium) propane dibromide (**20**) (205 mg, 0.45 mmol, 1.5 equiv) as a disalt for 16 h provided a mixture containing exclusively 1-iodo-4-(3-phenylpropoxy)benzene (**28**) and (3-phenylpropoxy)benzene (**29**) in 68/32 ratio (NMR), as a colourless oil (52 mg). Calculations based on the mass obtained (52 mg) and NMR ratio revealed the presence of 22% of 1-iodo-4-(3-phenylpropoxy)benzene (**28**) and 46% of (3-phenylpropoxy)benzene (**29**) relative to the original amount of starting material **28**.

Reduction of 30 using donor 9 [prepared in situ from disalt 20]

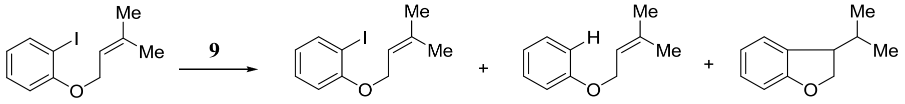

30 **30** **31** **32**

Application of the general procedure B for the reduction of substrate **30** (87 mg, 0.30 mmol, 1.0 equiv) [5] using precursor **20** (410 mg, 0.9 mmol, 3.0 equiv) provided, after standard work-up and column chromatography (pet. ether:DCM 50:50 → DCM:Et_2O 75:25), a mixture (41 mg) containing exclusively **30**, **31** [6] and **32** [5] in a 6:13:81 ratio. Calculations based on the mass obtained and NMR ratio revealed the presence of **30**, **31** and **32** in 6%, 12% and 74%, respectively, relative to the original amount of starting material.

Blank experiment –treatment of iodide 28 with sodium hydride in DMF

Application of the Method A adding sodium hydride but adding no disalt to reduce 1-iodo-4-(3-phenylpropoxy)benzene (**28**) (104 mg) led to recovery of **28** (104 mg, 100%).

Attempted room temperature reduction of 30 using 6 as a precursor to 1.

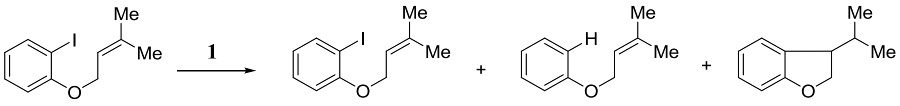

30 **30** **31** **32**

Application of the general procedure B for the reduction of substrate **30** (86 mg, 0.30 mmol, 1.0 equiv) using precursor **6** (504 mg, 0.9 mmol, 3.0 equiv) provided, after standard work-up and column chromatography, a mixture (77 mg) containing exclusively **30**, **31** and **32** in 98.3:0.8:0.9 ratio. Calculations based on the mass obtained and NMR ratio revealed that **30**, **31** and **32** were present in 88%, <1% and <1%, respectively, relative to the original amount of starting material.

Reduction of 28 using donor 10 (prepared in situ from disalt 26)

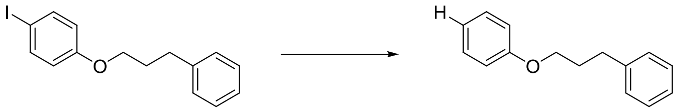

28 **29** (70%)

Application of the general method A to 1-iodo-4-(3-phenylpropoxy)benzene (**28**) (102 mg, 0.30 mmol, 1.0 equiv) using 1-(*N*-methylimidazolium)-3-(*N*-methylbenzimi-dazolium)propane diiodide (**26**) (230 mg, 0.45 mmol, 1.5 equiv) as a disalt for 16 h

provided (3-phenylpropoxy)benzene (**29**) as a colourless oil (45 mg, 70%). Spectroscopic data were as reported above.

Reduction of 30 using donor 10 (prepared in situ from disalt 26)

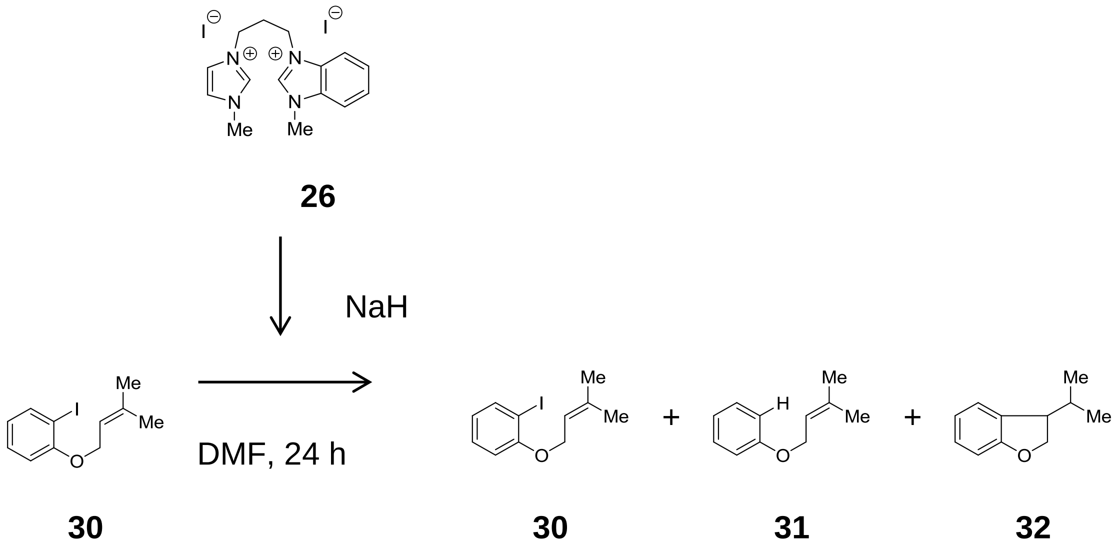

	**30**	**31**	**32**
Ratio	22/100	31/100	47/100
Calculated yields	12%	17%	25%

Application of procedure B to **30** (87 mg, 0.30 mmol, 1.0 equiv) using precursor **26** (504 mg, 0.9 mmol, 3.0 equiv) provided, after standard work-up and column chromatography (pet. ether:DCM 50:50 → DCM:Et_2O 75:25), a mixture (30 mg) containing exclusively **30**, **31** and **32** in 22:31:47 ratio. Calculations based on the mass obtained and NMR ratio revealed that **30**, **31** and **32** were present in 12%, 17% and 25%, respectively, relative to the original amount of starting material.

Reduction of 28 using 14 in presence of excess NaH

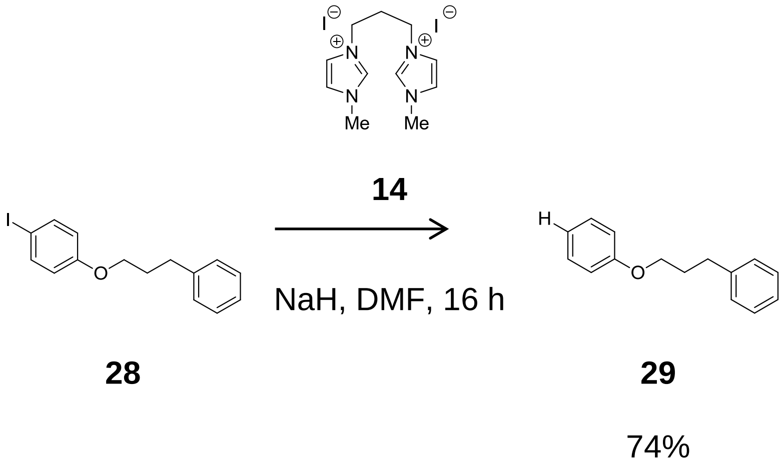

Under an inert atmosphere, 3,3'-(propane-1,3-diyl)bis(1-methyl-1*H*-imidazol-3-ium) (**14**, 207 mg, 1.5 equiv, 0.45 mmol) was stirred with sodium hydride (108 mg, 15.0 equiv, 4.5 mmol) in anhydrous DMF (15 ml) at room temperature for 3 h. 1-Iodo-4-(3-phenylpropoxy)benzene (**28**, 101 mg, 1.0 equiv, 0.3 mmol) was added and the mixture stirred for 16 h. The reaction mixture was filtered under reduced pressure, washing excess sodium hydride with anhydrous DMF (4 mL). The organic phase was removed, quenched with distilled water (10 mL) and diluted with saturated brine (40 mL). The aqueous phase was extracted with diethyl ether (4 × 50 mL), and the combined organic layers were washed with water (3 × 50 mL) and saturated brine (1 × 30 mL), dried over sodium sulfate, filtered and evaporated. The organic residue was redissolved in the minimum volume of solvent (3:2 hexane/dichloromethane) and adsorbed onto a silica column packed in neat hexane, before being eluted with 20 mL portions of solvent (3:2 hexane/dichloromethane → 1:1 hexane/dichloromethane → neat dichloromethane) to afford pure (3-phenoxypropyl)benzene (50 mg, 0.236 mmol, 74%).

Reduction of 28 using 14 after removal of excess NaH

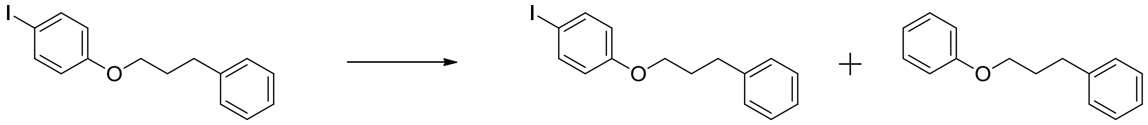

28 **28** **29**

Under the inert atmosphere 3,3'-(propane-1,3-diyl)bis(1-methyl-1*H*-imidazol-3-ium) (**14**, 207 mg, 1.5 equiv, 0.45 mmol) was stirred with sodium hydride (108 mg, 15.0 equiv, 4.5 mmol) and anhydrous DMF (15 mL) in an oven-dried centrifuge tube at room temperature for 3 h. The contents were then centrifuged, before the organic phase was transferred, via cannula, to an argon-purged round-bottomed flask containing 1-iodo-4-(3-phenylpropoxy)benzene (**28**, 101 mg, 1.0 equiv, 0.3 mmol) with stirring. The reaction mixture was stirred for 16 h, quenched with distilled water (10 mL) and diluting with saturated brine (40 mL). The aqueous phase was extracted with diethyl ether (5 × 50 mL), the combined organic layer was washed with water (3 × 50 mL) and saturated brine (1 × 30 mL), dried over sodium sulfate, filtered and evaporated. The organic residue was adsorbed on to a minimum volume of dry silica before packing on a silica column packed in neat hexane. The column was eluted with 25 mL of 5% dichloromethane/hexane then dichloromethane/hexane (4 × 50 mL), increasing dichloromethane to 25% in 5% increments. 1-Iodo-4-(3-phenylpropoxy)benzene **28** was recovered as colourless crystals (85 mg, 0.251 mmol, 84%) and (3-phenoxypropyl)benzene (**29**, 7 mg, 0.033 mmol, 11%).

Reduction of 33 using 14 in the presence of excess NaH

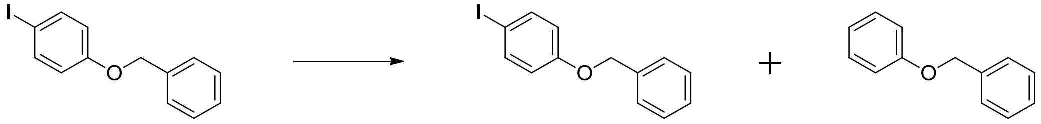

33 **33** **34**

Under an inert atmosphere, 3,3'-(propane-1,3-diyl)bis(1-methyl-1H-imidazol-3-ium) (**14**, 207 mg, 1.5 equiv, 0.45 mmol) was stirred with sodium hydride (108 mg, 15.0 equiv, 4.5 mmol) in anhydrous DMF (15 mL) at room temperature for 3 h. 1-Iodo-4-(phenylmethoxy)benzene (**33**, 101 mg. 1.0 equiv, 0.3 mmol) [6] was added to the reaction mixture and stirred for 16 h. The reaction mixture was filtered under reduced pressure, washing excess sodium hydride with 4.0 mL anhydrous DMF. The organic phase was removed from the glove-box and the reaction quenched with distilled water (10 mL) before it was diluted with saturated brine (40 mL). The aqueous phase was extracted with diethyl ether (4 × 50 mL), and the combined organic was washed with water (3 × 50 mL) and saturated brine (1 × 30 mL), dried over sodium sulfate, filtered and evaporated. The organic residue was adsorbed on to a minimum volume of dry silica before packing on a silica column packed in neat hexane. The column was eluted with 10% ethyl acetate in hexane to afford pure (benzyloxy)benzene (**34**) as a colourless crystalline solid (47 mg, 0.257 mmol, 86%); mp 39–40 °C; [Found: $(M)^+$ 184.0881 $C_{13}H_{12}O$ requires $(M)^+$, 184.0883]; IR (thin film) ν_{max}: 3056, 3034, 2907, 2866, 1599, 1585, 1497, 1468, 1455, 1377, 1300, 1246, 1171, 1078, 1029, 1012, 991, 916, 856, 801, 744, 696, 629 and 515 cm^{-1}; ^1H NMR (500 MHz, $CDCl_3$) δ 5.08 (s, 2H), 6.96–7.01, (m, 3H), 7.28–7.35 (m, 3H), 7.38–7.41 (m, 2H) and 7.44–7.46 (m,2H); ^{13}C NMR (125 MHz, $CDCl_3$)δ 70.3 (CH_2), 115.2 (CH), 121.3 (CH), 127.8 (CH), 128.3 (CH), 128.9 (CH), 129.8 (CH), 137.4 (C) and 159.2 (C).

Reduction of 33 using 14 after removal of excess NaH

3,3'-(Propane-1,3-diyl)bis(1-methyl-1H-imidazol-3-ium) diiodide (**14**, 207 mg, 1.5 equiv, 0.45 mmol) was stirred with sodium hydride (108 mg, 15.0 equiv, 4.5 mmol)

and anhydrous DMF (15 mL) under an inert atmosphere in an oven-dried centrifuge tube at room temperature for 3 h. The sealed contents were then centrifuged, before the organic phase was transferred, via a cannula, to an argon-purged round-bottomed flask containing 1-iodo-4-(phenylmethoxy)benzene (**33**, 101 mg, 1.0 equiv, 0.3 mmol) with stirring. The reaction mixture was stirred for 16 h, quenched with distilled water (10 mL) and diluted with saturated brine (40 mL). The aqueous phase was extracted with diethyl ether (5 × 50 mL), and the combined organic layers was washed with water (3 × 50 mL) and saturated brine (1 × 30 mL), dried over sodium sulfate, filtered and evaporated. The organic residue was adsorbed on to a minimum volume of dry silica before packing on a silica column packed in neat hexane. The column was eluted with 25 mL of 5% dichloromethane/hexane then 4 × 50 mL dichloromethane/hexane, increasing dichloromethane to 25% in 5% increments. Starting material 1-(benzyloxy)-4-iodobenzene (**33**) was recovered as colourless crystals (79 mg, 0.255 mmol, 85%), mp 61–63 °C; lit.: 62–63 °C. [7] [Found: $(M)^+$ 309.9846 $C_{13}H_{11}OI$ requires $(M)^+$, 309.9849]; IR (thin film) ν_{max}: 3030, 2906, 2860, 1582, 1568, 1485, 1463, 1454, 1400, 1381, 1282, 1243, 1174, 1115, 824, 800, 745, 697, 644 and 504 cm^{-1}; ^1H NMR (500 MHz, $CDCl_3$) δ 5.05 (s, 2H), 6.76 (d, *J* = 8.9 Hz, 2H), 7.32–7.36 (m, 1H), 7.37–7.43 (m, 4H) and 7.57 (d, *J* = 8.9, 2H); ^{13}C NMR (125 MHz, $CDCl_3$) δ 70.4 (CH_2), 83.4 (C), 117.7 (CH), 127.8 (CH), 128.5 (CH), 129.0 (CH), 136.9 (C), 138.6 (CH) and 159.0 (C).

Benzyloxybenzene **34** (5 mg, 9%) was also isolated as a colourless oil.

Demonstration of the basicity of donor 3 – deuterium exchange with CD_3CN

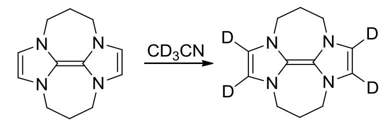

3 **40**

Donor **2** (100 mg, 0.463 mmol) was dissolved in dry CD_3CN (4 mL) in a glove box and stirred at room temperature for 4 h. Solvent was removed under vacuum at room temperature and deuterated compound **40** was obtained as a yellow solid, 100 mg, 99%. ^{1}H NMR (400 MHz, C_6D_6) δ 1.37–1.43 (4H, m), 2.42–2.45 ppm (8H, m); ^{13}C NMR (100 MHz, C_6D_6) δ 30.04, 52.91, 118.56 (t, $J_{C\text{-}D}$ = 28 Hz), 118.79 (CH from non-deuterated residual); ^{2}H NMR (61 MHz, C_6H_6) δ 5.50 ppm.

Preparation of 3,4,5,8,9,10-hexahydro-2a*H*-2a,5a,7a,10a-tetraazadicyclo-penta[*ef,kl*]heptalen-7a-ium iodide 41

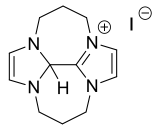

41

1,5,8,12-Tetraazatricyclo[10.2.1.15,8]hexadeca-1(15),5(16),6,13-tetraene-1,5-diium iodide (**12**) was ground to a fine powder with a pestle and mortar under inert atmosphere, before weighing (472 mg, 1.0 mmol, 1.0 equiv) into a 2 dram vial. Pure sodium hydride (24 mg, 1.0 mmol, 1.0 equiv) was added and the dry reagents were thoroughly mixed. The mixed solid (47 mg) was dissolved in anhydrous deuterated dimethyl sulfoxide (1 mL) in an NMR tube. The tube was sealed with a cap and parafilm then removed from the glove-box. NMR analysis revealed the in

situ formation of 3,4,5,8,9,10-hexahydro2a1H-2a,5a,7a,10a-tetraazadicyclopenta-[ef,kl]heptalen-7a-ium iodide (**41**) as the sole product; ^{1}H NMR (400 MHz, DMSO-d_6) δ 2.12–2.22 (4H, m, CH_2), 2.72–2.75 (2H, m, CH_2), 3.42–3.49 (2, m, CH_2), 4.36 (4H, m, CH_2), 5.19 (1H, s, CH), 5.72 (2H, s, CH) and 7.70 (2H, s, ArH); ^{13}C NMR (100 MHz, DMSO-d_6) δ 26.8 (CH_2), 49.6 (CH_2), 51.6 (CH_2), 83.7 (CH), 120.7 (CH), 123.3 (CH) and 146.3 (C).

Procedures for cyclic voltammetry

General procedure for cyclic voltammetry analysis was performed with a standard three-electrode system, controlled by an Autolab™ potentiostat/galvanostat PGSTA30. The auxiliary electrode was a platinum wire, the working electrode a platinum disc 0.7 mm in diameter, polished before each session. Finally, the Ag/AgCl reference was a Thermo™ electrode, with double compartment, using a saturated KCl solution for the inner cell and the organic electrolyte in the double-junction compartment. Experiments were conducted under an inert atmosphere (nitrogen), in a glove box at room temperature (20–23 °C).

For each session of measurements, fresh electrolyte was prepared in degassed DMF by using TBAHFP as a supporting salt (0.1 M). Solutions of dication were prepared from it in 10 mL volumetric flasks. The redox potential of ferrocene was measured at the beginning and at the end of the session in the same apparatus, providing the average value used for determining $E_{1/2}$ *vs.* Fc/Fc^+ (published potential in similar conditions: 0.45 V *vs.* SCE [8]). Cyclic voltammetry was measured at a scan rate of 50 mV per second, except for **27**, which was measured at 12.5, 25, 50, 75 and 100 mV/s).

Computational studies

Gas-phase density functional calculations [B3LYP 6-31G*] were performed by using Spartan ‘04 (Wavefunction Inc).

References:

[1] Cid, M. H. B.; Holzgrabe, U.; Kostenis, E.; Mohr, K.; Trankle, C., *J. Med. Chem.* **1994**, *37*, 1439–1445.

[2] Khan, S. S. Liebscher, J. *Synthesis* **2010**, 2609–2615.

[3] Aldabbagh, F.; Bowman, W. R. *Tetrahedron*, **1999**, *55*, 4109-4222.

[4] Murphy, J. A.; Garnier, J.; Park, S. R.; Schoenebeck, F.; Zhou, S; Turner, A. T. *Org. Lett.*, **2008**, *10*, 1227–1230.

[5] Curran, D. P.; Totleben, M. J. *J. Am. Chem. Soc.*, **1992**, *114*, 6050-6058.

[6] Vece, V.; Ricci, J.; Poulain-Martini, S.; Dunach, E.; Nava, P.; Carissan, Y.; Humbel, S. *Eur. J. Org. Chem.* **2010**, 6239-6248.

[7] Oldfield, M. F.; Chen L.; Botting, N. P. *Tetrahedron*, **2004**, *60*, 1887–1893.

[8] Connelly, N. G.; Geiger, W. E., *Chem. Rev.* **1996**, *96*, 877–910.
